# Association of physical activity and sedentary time with scoliosis screening positive in Chinese primary and secondary school students: a cohort study in Shanghai

**DOI:** 10.3389/fpubh.2025.1483007

**Published:** 2025-05-07

**Authors:** Liting Chu, Dongling Yang, Fengyun Zhang, Wenjuan Qi, Yuting Huang, Yanting Yang, Shuangxiao Qu, Shenglei Huang, Keyang Zheng, Chunyan Luo

**Affiliations:** Division of Child and Adolescent Health, Shanghai Municipal Center for Disease Control and Prevention, Shanghai, China

**Keywords:** students, scoliosis, physical activity, sedentary time, dynamic cohort, longitudinal analysis

## Abstract

**Background:**

The relationship between physical activity (PA), sedentary time (ST), and scoliosis screening positive (SSP) among Chinese students has not been extensively studied. This study aims to explore this association using data from the Shanghai Municipal Dynamic Cohort of Student Common Diseases (SMDCSCD).

**Methods:**

We conducted a repeated measures study over three waves (2021, 2022, and 2023). SSP was determined through physical examinations, while PA and ST data were collected via questionnaires. Sufficient moderate to vigorous PA (MVPA) was defined as more than 60 min per day, and excessive ST was classified as sitting for 4.5 h or more daily.

**Results:**

The study included 6,829 students with 19,673 observations. The prevalence of SSP ranged from 0.11% in Grade 1 to 2.77% in Grade 8 (*P*-trend < 0.001). Significant differences in ST (minutes/day) were found between the SSP and scoliosis screening negative (SSN) groups in the first 2 years (*p* = 0.026 and *p* = 0.023, respectively), but no significant differences were observed in total PA levels (MET-min/week) (*p* > 0.05). In a multivariable-adjusted model, ST of 4.5 h or more per day was positively associated with SSP (OR = 2.405, 95% CI: 1.323 to 4.374). No significant association was found between PA and SSP (*p* > 0.05).

**Conclusion:**

The prevalence of positive scoliosis screening increased with grade level. Longer sedentary time was significantly associated with a higher likelihood of positive scoliosis screening, while no significant association was found between physical activity levels and scoliosis screening outcomes. These findings suggest that reducing sedentary time may be important for scoliosis prevention in students, highlighting the need for targeted interventions to promote healthier daily habits.

## 1 Introduction

Scoliosis is characterized by abnormal curvature of the spine, torso, and chest, defined as a spinal curvature of ≥ 10° in the frontal plane (Cobb angle) with axial rotation. Idiopathic scoliosis, which accounts for approximately 80% of all scoliosis cases, has no known cause and is likely due to multiple factors (1). Adolescents over the age of 10 are particularly at risk for adolescent idiopathic scoliosis (AIS) (2).

The prevalence of AIS varies widely across different countries, with global rates estimated between 1 and 4%, making it the most common form of scoliosis (3). The spinal deformity associated with AIS often develops rapidly during early puberty, potentially impacting daily life and mental health. Symptoms may include back pain, postural asymmetry, and nerve root issues. If left untreated, scoliosis with a Cobb angle greater than 50° can continue to progress into adulthood, leading to impaired pulmonary and cardiovascular function, and in severe cases, thoracic insufficiency syndrome (4). Studies have shown that an increased Cobb angle can adversely affect respiratory function, with pulmonary impairment occurring earlier than previously anticipated in AIS patients (5, 6). Furthermore, when the curve reaches between 20° and 120°, the spinal Cobb angle is inversely related to pulmonary function (7), posing a significant health risk to young people. Early screening and identification of risk factors are therefore crucial in preventing severe scoliosis progression.

Emerging evidence suggests that insufficient physical activity (PA) is a potential risk factor for the development of AIS in school-age children (8–10). This is particularly evident in those suspected of having AIS. Glavaš et al. (8) found that students with positive results on the forward bend test were less physically active than their peers without scoliosis, and that a higher prevalence of suspected AIS was observed among inactive or recreationally active schoolchildren compared to those engaged in organized sports. Similarly, Oba et al. (11) reported that improvements in physical flexibility after scoliosis surgery had a positive effect on respiratory function. Cong et al. (12) also found that dynamic cardiopulmonary capacity was correlated with quality of life in AIS patients, suggesting that exercise might enhance quality of life in these individuals.

Sedentary behavior is another critical aspect of PA that is associated with long-term health outcomes (13). One study documented that poor sitting posture was linked to thoracic hyperkyphosis in men as well as head anteriorization and pelvic retroversion in women (14). Zhu et al. (15) presented that AIS was attributable in part to excessive sedentary time (ST), which is echoed by an accelerometer-based assessment focused on adolescents and highlighted the negative impact of prolonged sitting, especially when combined with decreased PA (16).

However, some studies have reported conflicting findings. A cross-sectional study found no significant differences in moderate activity levels or ST between adolescents with and without AIS (17). Likewise, no substantial difference in the incidence of AIS was observed between athlete and non-athlete adolescents, with no change in the degree of scoliotic curvature among those actively participating in sports (18). While students may have more regular PA patterns than adults due to school schedules and compulsory physical education, it remains unclear whether these patterns consistently affect spinal health across different educational stages.

Despite the existing body of knowledge, the relationship between PA, ST, and scoliosis in a large sample of children has not been fully explored, particularly in the Chinese population across various ages. This study aims to build on previous research by analyzing data from a representative cohort. We assessed trends in the prevalence of scoliosis screening positive (SSP) among different grades, stratified by gender, over 3 years to monitor students’ spinal health. Our focus was on PA at the nationally recommended level and ST at a risk threshold to establish a strong longitudinal relationship between physical behavior and spinal abnormalities in primary and secondary school students. Additionally, we examined potential modifiers between these factors and SSP. The findings of this study will provide valuable insights for health providers, policymakers, and healthcare professionals to develop strategies for preventing the occurrence and progression of scoliosis in children, improving scoliosis management among students.

## 2 Methods

### 2.1 Study design and participants

Participants of this study were from the “Shanghai Municipal Dynamic Cohort of Student Common Diseases (SMDCSCD), 2021–2023,”(42) an ongoing, large-scale, municipal-level longitudinal survey initiated in 2021. The primary objective of this cohort is to monitor the prevalence, progression, and associated risk factors of common student health conditions, including adolescent myopia, obesity, scoliosis, and dental caries, in a school-based setting.

The study employed a multi-stage stratified cluster sampling strategy to ensure a representative sample of primary and secondary school students across all 16 districts of Shanghai. In the first stage, schools were randomly selected from each district based on school size and type to ensure broad representation. In the second stage, over 300 students from Grade 1 (primary school) and Grade 6 (secondary school) were recruited per district, forming the baseline cohort. These students have been followed up annually with repeated measurements through standardized on-site physical examinations and structured online questionnaires. The follow-up process involved tracking the initial cohort as they progressed through school grades. In 2022, students in Grade 2 and Grade 7 were reassessed, and in 2023, the follow-up extended to Grade 3 and Grade 8. By 2023, a total of 7,347 students had participated in the study, contributing 20,325 recorded observations across six different grade levels. For this specific analysis, we focused on students with valid scoliosis screening data (*n* = 7,341). To ensure longitudinal comparability, we excluded students with only one recorded visit, resulting in a final analytic sample of 6,829 students with 19,673 observations distributed across 16 districts of Shanghai.

The study was approved by the Ethics Committee of the Shanghai Municipal Center for Disease Control and Prevention (2022-13). Written informed consent was obtained from the parents or legal guardians of all participating students before enrollment in the study.

### 2.2 Screening of scoliosis positive

Scoliosis screening was an integral component of the standardized physical examinations conducted annually in schools. The screening was performed by highly trained medical practitioners from local community healthcare centers, ensuring consistency and accuracy. Examinations took place in well-lit rooms specifically designated for medical assessments and equipped with professional instruments, including examination beds and scoliosis meters (Jiansheng, China), to facilitate precise measurements.

The screening procedures followed established national guidelines. In 2021, the assessment was conducted in accordance with the National Standard for Detecting Abnormal Spinal Curvature in Children and Adolescents (GB/T 16133—2014) (19). The subsequent 2 years used an updated version of this standard, the Technical Guide for Prevention and Control of Spinal Curvature Abnormality in Children and Adolescents (20). The screening process involved multiple steps to ensure thorough evaluation, beginning with a general back examination where each student was assessed from a posterior view while standing to identify visible asymmetries or postural irregularities. Students then underwent the Adams Forward Bend Test, where they bent forward at the waist with straight knees to assess back symmetry, particularly rib or lumbar prominence, which may indicate spinal curvature abnormalities. A scoliosis meter was used to quantify trunk rotation and detect angular deviations in the spine, with measurements exceeding a predefined threshold considered indicative of potential scoliosis. Students who exhibited abnormal findings in these initial assessments underwent a follow-up spinal movement test to confirm the presence of spinal curvature irregularities. Those identified as having suspected scoliosis were refered for further medical assessment and follow-up. The detailed screening process is illustrated in Supplementary Figure S1.

### 2.3 Physical activity and sedentary time

We used the self-reported International Physical Activity Questionnaire short form (IPAQ-SF) to determine PA levels and ST (21), which has been validated for estimating PA levels in Chinese children (22). Secondary school students completed the questionnaire themselves, while primary school students’ responses were provided by their parents or primary caregivers. Respondents were asked to recall the frequency and duration of three types of activities (vigorous intensity, moderate intensity, and walking) over the past 7 days, as well as the average daily time spent on sedentary behaviors.

Total weekly PA was calculated in Metabolic Equivalent Task minutes per week (MET-min/week), with MET values assigned as follows: vigorous-intensity activities (8 METs), moderate-intensity activities (4 METs), and walking (3.3 METs) (23). According to national guidelines on PA for children and adolescents aged 6 to 17 years (24), achieving more than 60 min of moderate to vigorous physical activity (MVPA) per day was considered sufficient.

ST was reported in hours per day (h/day) and categorized as excessive if students sat for more than 4.5 h daily, a threshold associated with an increased risk of cardiovascular disease (25). Reported ST exceeding 1,000 min per day was treated as missing data.

### 2.4 Covariates

We included a range of covariates in our analysis, such as demographic information (gender, age, grade, ethnicity, residence, BMI, academic performance, parents’ education, family income, and family history of AIS), eye movement behaviors (e.g., resting 10 min after 30–40 min of continuous eye use, maintaining over 40 cm distance from smartphones and tablets, and adherence to proper reading and writing posture), and spinal health-related behaviors (e.g., consistent self-monitoring of sitting and standing posture, adjusting desk and seat height at home, and parental opinions on the suitability of seating arrangements).

Specifically, eye rest and screen distance were categorized as “always,” “sometimes,” and “never.” Adherence to proper reading and writing posture (one-punch, one-foot, and one-inch) was a binary variable, with “up to the standard” indicating adherence to posture guidelines more than 70% of the time. Self-monitoring of posture was categorized as either “constant keep” (always maintaining correct posture or self-reminding) or “non-constant keep” (prioritizing comfort).

### 2.5 Statistical analysis

Descriptive statistics are shown as mean with standard deviation or median (interquartile range [IQR]), relying on the Kolmogorov–Smirnov test. Categorical variables were expressed as number (percentage). The trend analyses for prevalence in SSP over grades were conducted with Cochran-Armitage trend test. Differences in total PA and ST between the scoliosis screening negative (SSN) and SSP groups were assessed using the Mann–Whitney U test.

Given the longitudinal design with repeated measurements, generalized estimating equations (GEE) (26) were used to evaluate the influence of PA and ST on SSP, with a binomial distribution family and an independent working correlation structure. Before modeling, we used Spearman correlation matrices to check for collinearity among variables (Supplementary Table S1 and Supplementary Figure S2), with a threshold correlation coefficient of less than 0.5. Due to a relatively high correlation between age and BMI (*r_s_* = 0.506), age was excluded from further models. We defined models with distinct levels of adjustment. Model 1 included PA and ST adjusted for the year. Model 2 additionally controlled for demographic information. Model 3 further adjusted for eye movement-related behaviors, and Model 4 was a multivariable-adjusted model that also included spinal health-related behaviors.

Sensitivity analyses were performed to validate the robustness of the findings. Missing values for ST were imputed using multiple imputation, generating five imputation models that included variables likely to predict missing values. Estimates from each imputation were pooled using Rubin’s rules (27). Subgroup analyses examined potential effect modifiers, such as place of residence, educational stage, gender, academic performance, adherence to reading and writing posture guidelines, piano playing, and home desk and seat height adjustments, with year as a confounder.

**Figure 1:**
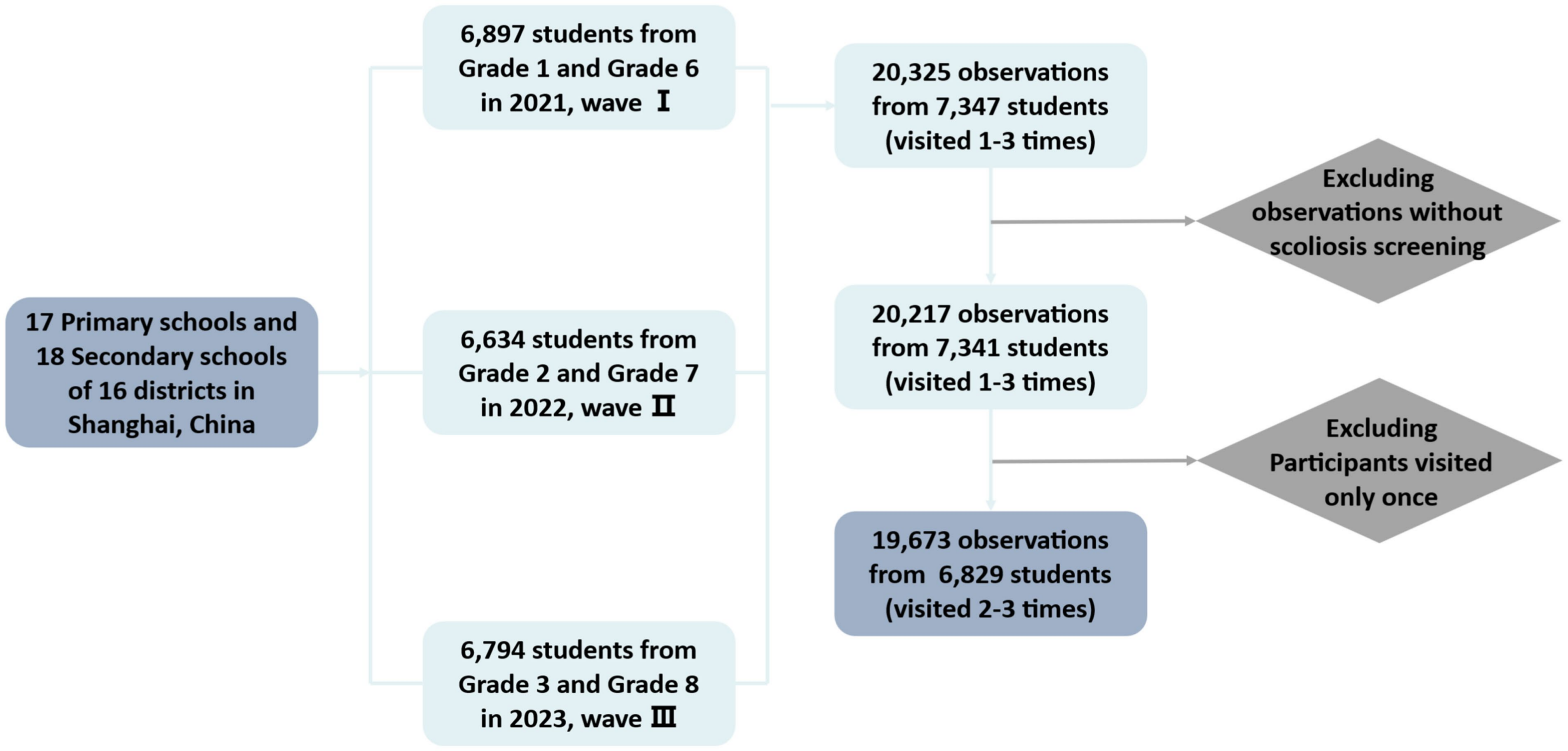
Flowchart of sample inclusion and exclusion.

**Figure 2:**
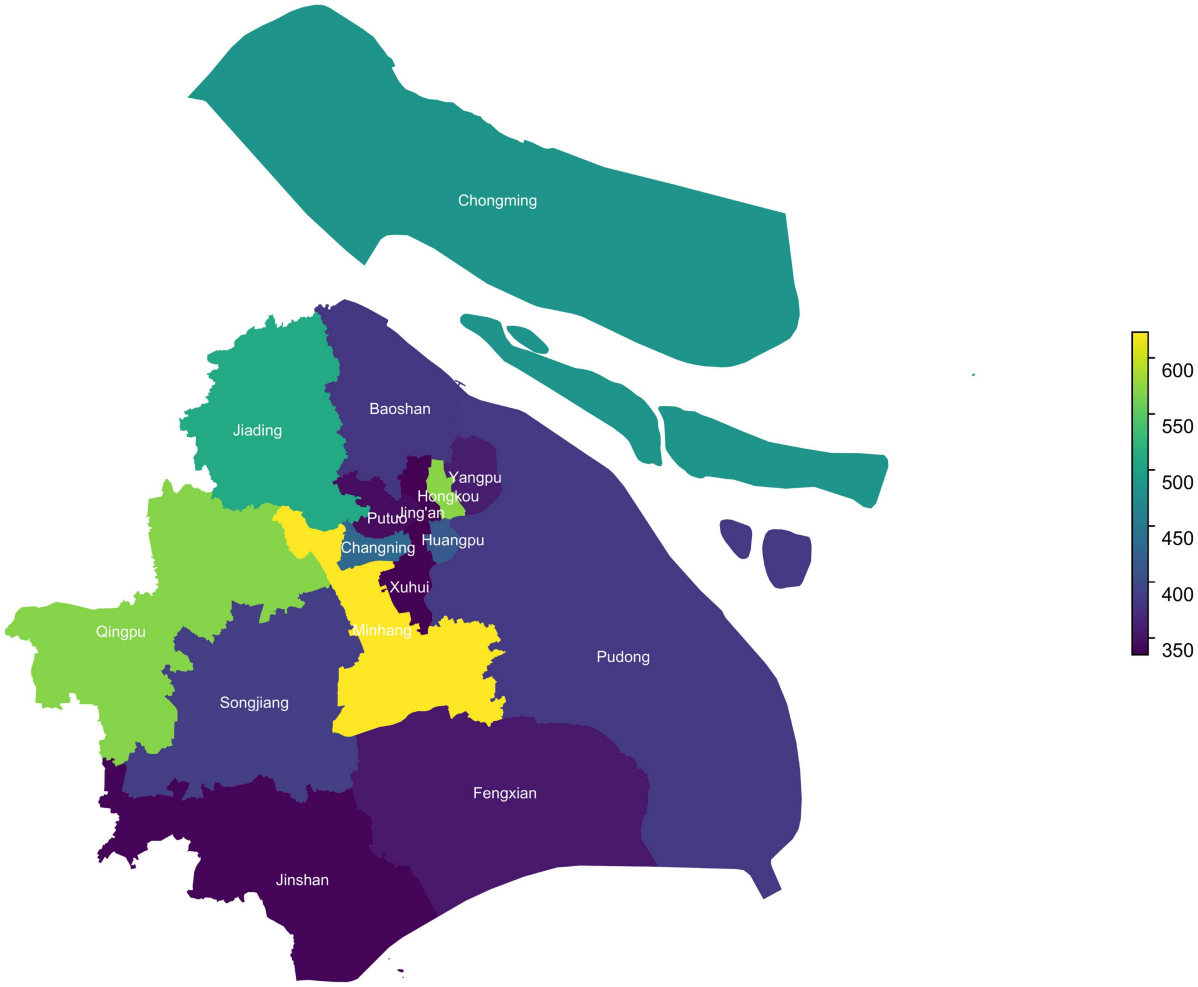
The map of participants’ geographic distribution in Shanghai (2021–2023).

Two-sided *p*-values less than 0.05 were considered statistically significant. Analyses were run in R, version 4.3.2 (R Foundation for Statistical Computing, Vienna, Austria) and Python, version 3.11.5 (see Figures 1, 2).

## 3 Results

Table 1 presents the sociodemographic characteristics and family history of adolescent idiopathic scoliosis (AIS) among the 6,829 students studied, with 3,640 (53.3%) in primary school and 3,189 (46.7%) in secondary school. Longitudinal data from the baseline and two subsequent follow-up waves can be found in Supplementary Table S2.

**Table 1:** Constant variables of all studied subjects.

Variables	No. (%)
Total number of Subjects	6,829
Place of residence	
Urban	2,794 (40.9)
Rural	4,035 (59.1)
Education stage	
Primary school	3,640 (53.3)
Secondary school	3,189 (46.7)
Gender	
Boys	3,453 (50.6)
Girls	3,376 (49.4)
Ethnic groups	
Han	6,704 (98.2)
Non-Han	125 (1.8)
Family AIS history	
No	6,805 (99.7)
Yes	11 (0.2)
Missing	13 (0.2)

**Figure 3:**
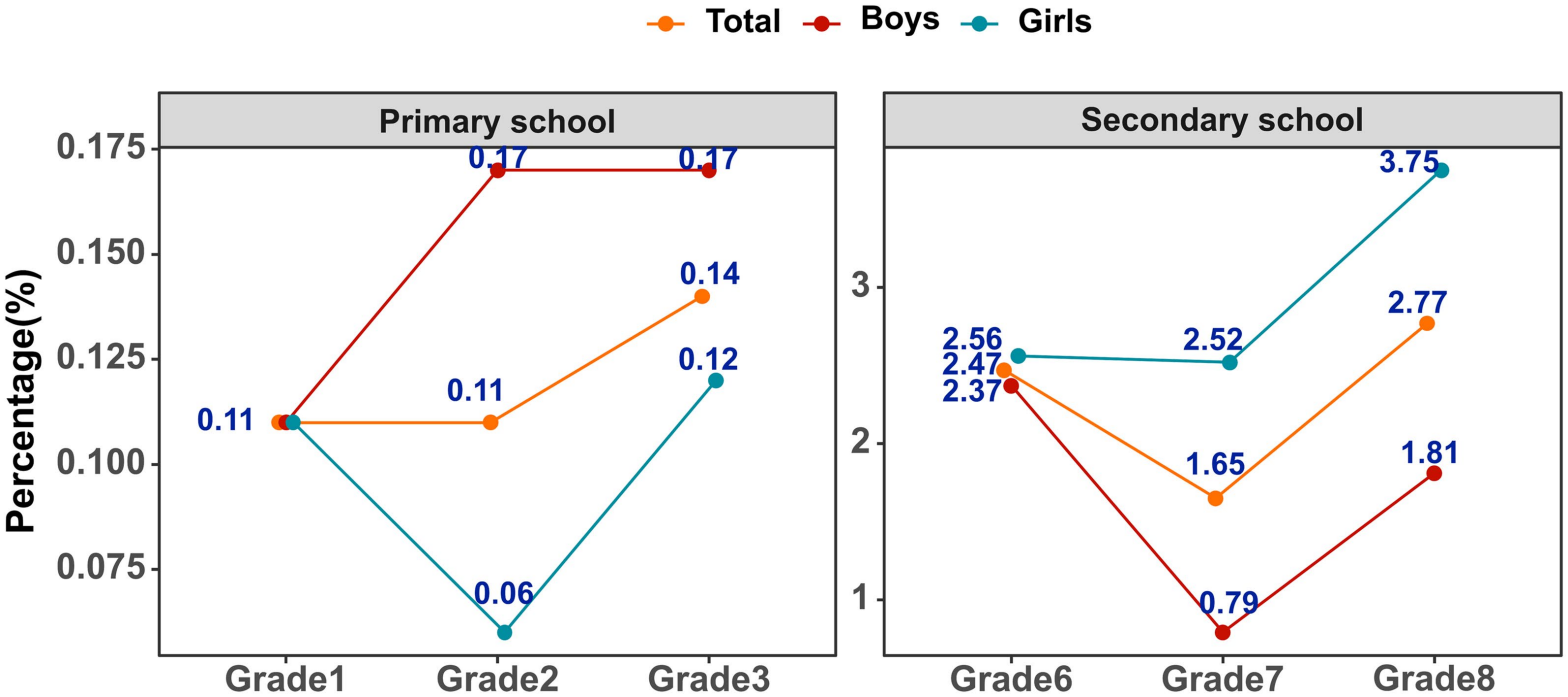
Prevalence of scoliosis screening positive over 3 years by grade and gender, respectively.

Figure 3 illustrates the changes in the prevalence of SSP over the 3 years of the study. The overall prevalence of SSP was 1.13%, ranging from a low of 0.11% among Grade 1 students to a high of 2.77% among Grade 8 students (*P*-trend < 0.001). This increasing trend was consistent across all six grades when stratified by gender (*P*-trend < 0.001 for both boys and girls). However, when the data were divided into two educational stages (primary and secondary), no significant trend differences were observed (all *P*-trend > 0.05; see Supplementary Table S3 for details). The highest prevalence of SSP was observed among Grade 8 girls, with a rate of 3.75%. The distribution of scoliosis severity among SSP students is shown in Supplementary Figure S3.

**Figure 4:**
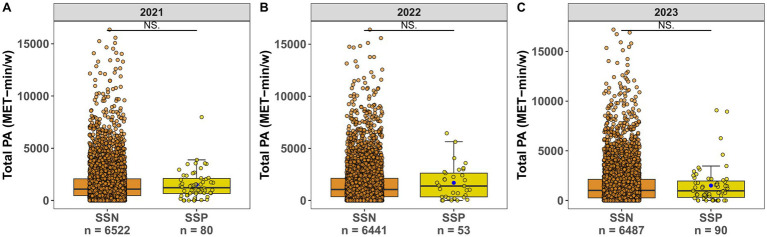
**(A)** Total physical activity (MET-min/w) in 2021. **(B)** Total physical activity (MET-min/w) in 2022. **(C)** Total physical activity (MET-min/w) in 2023.

**Figure 5:**
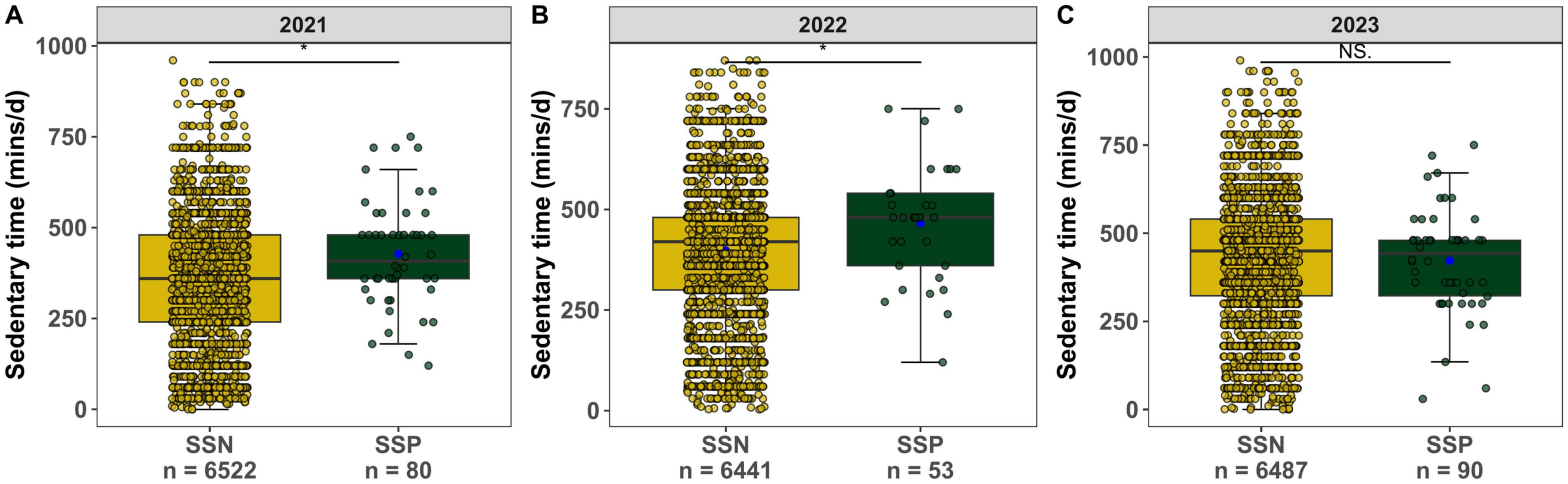
**(A)** Sedentary time (mins/d) between groups in 2021 **(B)** Sedentary time (mins/d) between groups in 2022. **(C)** Sedentary time (mins/d) between groups in 2023.

We compared the difference in total PA level (MET-min/week) and ST (mins/day) between SSN and SSP students over 3 years. In the whole sample, reported total PA level showed a median of 1053.00 MET-min/week (interquartile range: 372.00 to 2118.00 MET-min/week), and no significant difference was observed between groups, which was true of the results in each year (*p* > 0.05, Figures 4A–C). By contrast, those of SSP had higher sitting duration than their SSN counterparts; median [IQR] of 480.00[352.50–510.00] versus 420.00[300.00–480.00] mins/day respectively, *p* = 0.026. The same pattern could be found during the first consecutive 2 years (*p* = 0.026, *p* = 0.023 in Figures 5A,B respectively), whereas the third year saw no marked statistical difference of ST in the two groups (*p* = 0.710, Figure 5C). The concrete values of total PA level and ST are listed in Supplementary Table S4.

Table 2 examines the relationship between SSP, PA, and ST. Across four different models, excessive ST (more than 4.5 h/day) consistently showed a detrimental effect on SSP, with an odds ratio (OR) of 2.405 (95% CI: 1.323 to 4.374) compared to less than 4.5 h/day. No significant relationship was found between insufficient PA and SSP (multivariable-adjusted model, OR: 1.237, 95% CI: 0.727 to 2.102). Furthermore, no significant association between ST and SSP was found in Model 1 when stratified by education stage (*p* > 0.05; see Supplementary Table S5).

**Table 2:** Estimated associations of insufficient MVPA and excessive sedentary time with scoliosis screening positive.

Variables	Model 1 (*n* = 10,726)		Model 2 (*n* = 10,359)		Model 3 (*n* = 10,355)		Model 4 (*n* = 10,353)	
	OR (95%CI)	*p*	OR (95%CI)	*p*	OR (95%CI)	*p*	OR (95%CI)	*p*
MVPA								
Sufficient	ref.		ref.		ref.		ref.	
Insufficient	1.208 (0.729 to 2.000)	0.463	1.232 (0.726 to 2.091)	0.439	1.232 (0.726 to 2.091)	0.439	1.237 (0.727 to 2.102)	0.433
Sedentary time								
<4.5 h/d	ref.		ref.		ref.		ref.	
≥4.5 h/d	2.402 (1.351 to 4.271)	0.003	2.405 (1.321 to 4.380)	0.004	2.410 (1.325 to 4.384)	0.004	2.405 (1.323 to 4.374)	0.004

**Figure 6:**
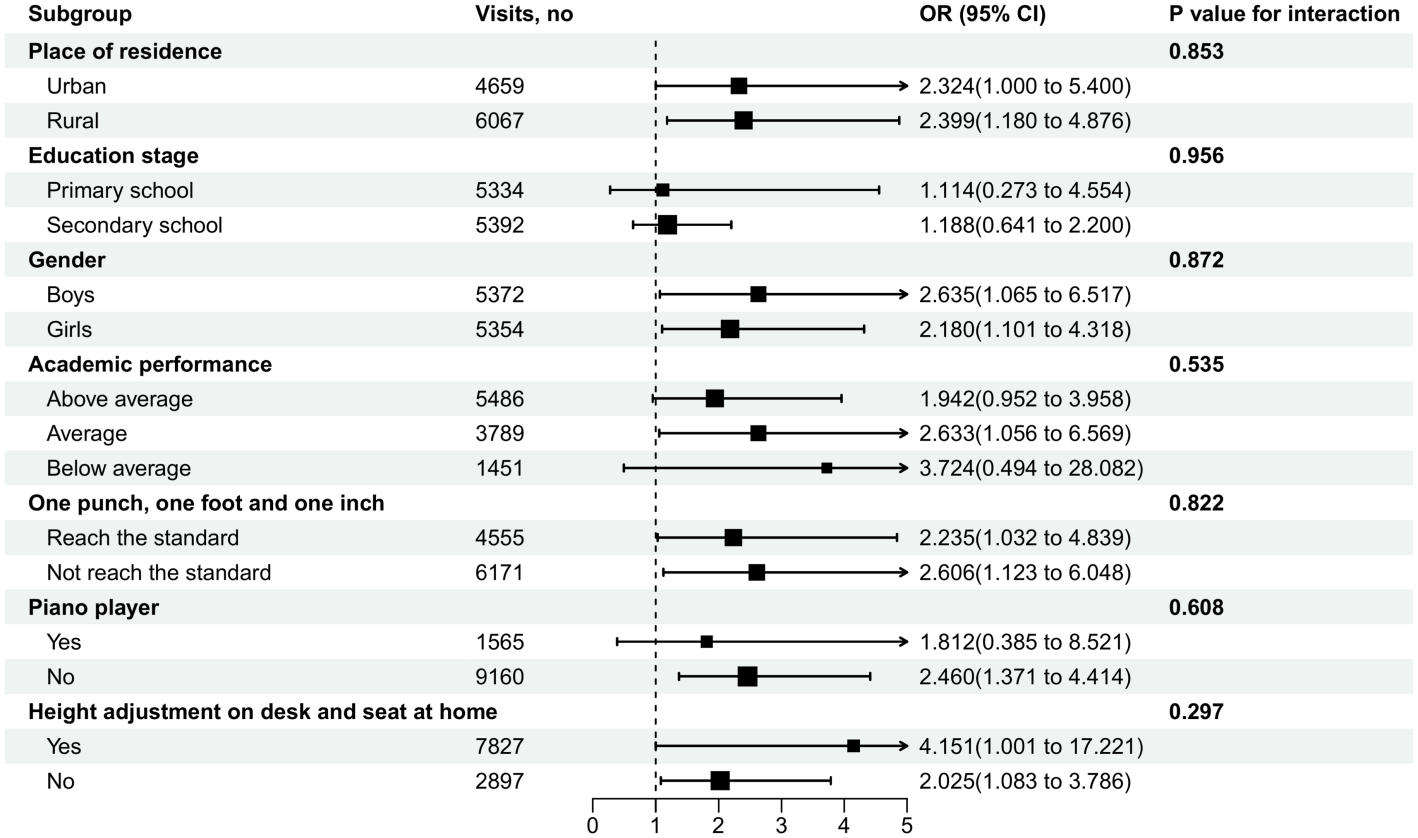
Subgroup-specific associations of sedentary time with scoliosis screening positive.

Subgroup analysis on ST-SSP association stratified by demographic, eye movement, and spinal health factors is outlined in Figure 6. Despite the higher risk of SSP among students in rural area, average academic performance, non-piano player, and height adjustment on desk and seat at home, there was no statistically significant interaction between relevant factors and ST (*p* > 0.05).

The results of imputations for missing ST data, conducted using four GEE models, revealed similar findings (Table 3). Excessive ST (>4.5 h/day) remained positively associated with SSP (OR: 2.312, 95% CI: 1.322 to 4.044).

**Table 3:** Estimated associations of insufficient MVPA and excessive sedentary time with scoliosis screening positive (imputed data).

Variables	Model 1 (*n* = 19,673)		Model 2 (*n* = 18,635)		Model 3 (*n* = 18,629)		Model 4 (*n* = 18,626)	
	OR (95%CI)	*p*	OR (95%CI)	*p*	OR (95%CI)	*p*	OR (95%CI)	*p*
MVPA								
Sufficient	ref.		ref.		ref.		ref.	
Insufficient	1.482 (0.907 to 2.423)	0.117	1.445 (0.867 to 2.408)	0.158	1.459 (0.874 to 2.437)	0.148	1.472 (0.881 to 2.461)	0.140
Sedentary time								
<4.5 h/d	ref.		ref.		ref.		ref.	
≥4.5 h/d	2.464 (1.381 to 4.396)	0.006	2.394 (1.378 to 4.159)	0.003	2.320 (1.329 to 4.052)	0.005	2.312 (1.322 to 4.044)	0.005

## 4 Discussion

This study is the first to present a three-year trend in the prevalence of SSP across two distinct educational stages, and it highlights a significant link between ST and SSP based on data from an ongoing municipal dynamic cohort. We observed that students who did not test positive for scoliosis exhibited reduced ST compared to those suspected of spinal curvature, a trend consistently presented across all subgroups in our study. Surprisingly, no significant association was found between PA and SSP, with the rate of MVPA meeting national standards being less than 15% over 3 years.

One notable finding consistent with previous research is the higher incidence of scoliosis among adolescent girls (28), with the peak prevalence of presumed scoliosis reaching 3.75% in the 8th grade. Several studies have reported that girls, particularly during the teenage period (29) or preadolescence (30) with potential physical signs of spinal deformity such as asymmetrical shoulder height, flank asymmetry, etc., or even progressive scoliosis. Concomitantly, the American Academy of Orthopaedic Surgeons estimates that girls are ten times more likely to develop AIS than boys before the age of ten. Additionally, when a girl is diagnosed with scoliosis, she is roughly ten times more likely to experience progression than boys of a similar age (31).

The underlying reasons for this gender disparity remain unclear (32). Some theories suggest a connection to the body’s autonomic nervous system, which influences puberty and skeletal growth through hypothalamic neuroendocrine control, including hormone levels (33). Studies have also explored the role of leptin, a hormone crucial for regulating bone and energy metabolism in children, in the mechanisms of scoliosis. Research has shown discrepancies in leptin and soluble leptin receptors between AIS girls and healthy controls (34), with meta-analyses confirming these findings (35). However, our study did not include laboratory examinations due to the large sample size and instrument limitations, which should be addressed in future research.

The relationship between PA and scoliosis has been inconsistent in previous studies. According to de Assis et al. (9), PA appears to be a protective factor for schoolchildren with scoliosis where IPAQ and a questionnaire were used to estimate the PA level. The same result could also be found in a prospective cohort study, where the results presented that reduced physical ability and activity at an early age were associated with increased risk of scoliosis onset during juvenile stages (10). Contrary to these findings, an inconspicuous correlation between exercise and AIS (18) as well as postural disorders was documented in some studies, which concurred with this research that students of SSP were as equally active as their peers of SSN. An explanation for this finding is the different approach to physical measurement. The 9-item IPAQ records PA levels in terms of self-reported or caregiver-filled MVPA and walking without concrete sports type, which may lead to the differing associations. McMaster’s findings showed that progressive AIS was positively related with indoor heated swimming pools, whereas negatively associated with participation in dance, skating, gymnastics, karate, and football or hockey classes (36), which means the type of sports could make a huge difference to the scoliosis progression. Plus, general PA has been proven to exert a beneficial effect on bone density (37). However, the existing national standard (20) on the control and prevention of adolescent scoliosis has similar suggestions on physical exercise as the national and the World Health Organization guidelines, it is noted that further studies on high-impact activity should be specifically addressed.

Long-term sitting (i.e., ≥4.5 h/d) was observed in those with presumed scoliosis. The primary cause is the influence of sitting posture on the spine. Araújo et al. (14) demonstrated the association between poor sitting posture and spinal alterations in adolescents. When individuals maintain a sitting posture for a long time, especially sitting incorrectly, the spine may be subjected to abnormal pressure distribution, which may cause or aggravate scoliosis. An alternative explanation for this association is the unbalanced muscle function. A related study found that patients with progressive AIS had imbalanced paraspinal muscular activity when compared to healthy individuals, suggesting that asymmetric dysregulation of the musculature may be the underlying cause (38). An imbalance in the muscles surrounding the spine may result from prolonged sitting. Overuse can cause some muscle groups to become stiff and other muscle units to relax. This misalignment of muscles may exacerbate scoliosis (39). Moreover, sitting for long periods restricts the movement of the body, resulting in insufficient stretching and exercise of the spine and surrounding muscles, which may exacerbate scoliosis by making it more difficult for the spine to retain its natural physiological curvature (40). According to our results, the proportion of total participants sitting for more than 4.5 h per day was close to four-fifths (79.7%), raising concerns about potential spinal health issues. However, the association between ST and presumed scoliosis changed when stratified by educational stage, possibly indicating that age is a crucial factor in the development of scoliosis. As students grow older, particularly in secondary school, their academic workload typically increases, which may lead to more time spent sitting and studying. This increased sedentary behavior could partially explain the observed association between ST and SSP, as older students tend to have higher levels of sedentary time compared to younger students. The positive correlation between age and sitting time, along with the relatively small number of SSP cases in each educational period, may have influenced these results. Therefore, it is essential to distinguish whether the relationship between ST and SSP is due to sedentary habits themselves or if it is primarily a reflection of age-related changes in academic and daily routines. Plus, further research is needed to determine whether the observed associations are causal and to develop theory-based prevention and intervention strategies that can be integrated into adolescent scoliosis management to reduce sedentary behavior.

### 4.1 Strength and limitations

This study sheds light on the association between physical behavior and SSP using data from a three-year longitudinal cohort, addressing a gap in previous Chinese research that often lacked follow-up data on this relationship (41). Our study’s strengths include a low overall drop-out rate (7.1%), adherence to national standards for physical examinations, and comprehensive data collection during scoliosis screenings, all of which enhance the robustness of our findings. However, the study has limitations due to the restricted instruments used. Key measurements, such as laboratory examinations, clinical diagnoses, and precise physical behavior assessments, were not conducted. To improve future research, the use of wearable devices and specific sports questionnaires is recommended for more accurate measurement of PA and monitoring of ST.

## 5 Conclusion

In summary, this study observed an upward trend in the prevalence of scoliosis screening positive (SSP) across different grades within a three-year longitudinal cohort of a representative municipal sample of students. The findings provide evidence that prolonged sitting is positively associated with SSP, while no significant link was found between insufficient physical activity and presumed scoliosis. These results underscore the need for future research that further quantifies the impact of sedentary behavior and physical activity on scoliosis development. Specifically, future studies should aim to measure sedentary time, physical activity levels, and sport types with greater precision to better understand how these factors influence spinal health. Additionally, research should consider the potential confounding effects of age, academic workload, and lifestyle changes as students grow older, as these may contribute to the observed associations. Understanding these relationships is critical for developing targeted interventions to mitigate spinal health issues in adolescents and ensure their long-term well-being.

## Data Availability

The original contributions presented in the study are included in the article/Supplementary material, further inquiries can be directed to the corresponding author.
