# Supplementary material for: Association of physical activity and sedentary time with scoliosis screening positive in Chinese primary and secondary school students: a cohort study in Shanghai
Source: Front Public Health. 2025 May 7;13:1483007. doi: 10.3389/fpubh.2025.1483007 (PMC12092463; doi:10.3389/fpubh.2025.1483007)

Supplementary Material

Table S1. The correlation of independent variables

	**1**	**2**	**3**	**4**	**5**	**6**	**7**	**8**	**9**	**10**	**11**	**12**	**13**	**14**	**15**	**16**	**17**	**18**
1	1.000																	
2	0.077**	1.000																
3	0.293**	-0.009	1.000															
4	-0.006	0.018*	-0.023*	1.000														
5	-0.023**	0.005	-0.006	-0.023**	1.000													
6	0.506**	-0.040**	0.141**	-0.009	-0.013	1.000												
7	0.033**	0.006	0.018	-0.022**	-0.005	0.011	1.000											
8	0.149**	0.051**	0.025**	0.067**	-0.010	0.134**	0.009	1.000										
9	-0.109**	-0.018*	0.016	-0.217**	0.034**	-0.112**	0.006	-0.215**	1.000									
10	-0.124**	-0.011	0.042**	-0.222**	0.012	-0.120**	-0.002	-0.221**	0.588**	1.000								
11	-0.121**	0.055**	-0.036**	0.105**	0.006	-0.037**	-0.031**	0.212**	-0.202**	-0.195**	1.000							
12	0.071**	0.084**	0.068**	-0.025**	-0.005	0.041**	0.026**	0.166**	-0.006	0.001	0.082**	1.000						
13	0.062**	0.047**	0.053**	-0.001	-0.000	0.031**	0.028**	0.117**	-0.017*	-0.005	0.079**	0.328**	1.000					
14	-0.021**	0.071**	0.041**	-0.004	0.016*	-0.014*	0.014*	0.119**	-0.006	0.007	0.087**	0.351**	0.319**	1.000				
15	0.026**	0.009	-0.000	0.062**	-0.002	0.048**	-0.004	0.127**	-0.157**	-0.171**	0.122**	0.031**	0.034**	0.016*	1.000			
16	-0.054**	0.076**	0.022*	-0.037**	0.005	-0.048**	-0.002	0.063**	0.041**	0.057**	0.055**	0.154**	0.106**	0.177**	0.003	1.000		
17	-0.147**	-0.019**	-0.052**	-0.064**	0.004	-0.088**	-0.025**	-0.082**	0.058**	0.065**	-0.027**	-0.141**	-0.128**	-0.125**	-0.033**	-0.055**	1.000	
18	0.106**	0.001	0.039**	-0.006	0.010	0.064**	0.013	0.034**	0.020**	0.026**	0.014*	0.095**	0.104**	0.091**	-0.005	0.039**	-0.190**	1.000

Note:1.Age (yrs); 2.MVPA; 3.Sedentary time; 4.Place of residence; 5.Ethnic groups; 6.BMI (kg/m^2); 7.Family AIS history; 8.Academic

performance; 9.Father's education; 10.Mother's education; 11.Family income status; 12.Rest after 10-min continuous eye use; 13.Over 40 cm distances from electronic devices; 14.One punch, one foot and one inch; 15.Piano player; 16.Self-requirements on sitting and standing posture; 17.Height adjustment on desk and seat at home; 18.Parents' view on the suitability of seat and desk for child;

Table S2. Longitudinal variables of the studied subjects at baseline and two subsequent waves.

Variables	Total, n (%)	2021	2022	2023
Total number of Visits	19,673	6,602	6,494	6,577
Grade				
Grade1	3521 (17.9)	3521 (53.3)	0 (0.0)	0 (0.0)
Grade2	3518 (17.9)	0 (0.0)	3518 (54.2)	0 (0.0)
Grade3	3507 (17.8)	0 (0.0)	0 (0.0)	3507 (53.3)
Grade6	3081 (15.7)	3081 (46.7)	0 (0.0)	0 (0.0)
Grade7	2976 (15.1)	0 (0.0)	2976 (45.8)	0 (0.0)
Grade8	3070 (15.6)	0 (0.0)	0 (0.0)	3070 (46.7)
Age (yrs)	9.93±2.63	8.94±2.50	9.93±2.50	10.94±2.50
BMI (kg/m2)	18.53±4.05	17.87±3.75	18.69±4.07	19.04±4.24
Academic performance				
Above average	9190 (46.7)	3420 (51.8)	2824 (43.5)	2946 (44.8)
Average	7427 (37.8)	2577 (39.0)	2477 (38.1)	2373 (36.1)
Below average	2870 (14.6)	552 (8.4)	1074 (16.5)	1244 (18.9)
Missing	186 (0.9)	53 (0.8)	119 (1.8)	14 (0.2)
Father's education				
Below junior college	4286 (21.8)	1540 (23.3)	1438 (22.1)	1308 (19.9)
Junior college/college	12612 (64.1)	4331 (65.6)	4211 (64.8)	4070 (61.9)
Above college	2051 (10.4)	678 (10.3)	676 (10.4)	697 (10.6)
Missing	724 (3.7)	53 (0.8)	169 (2.6)	502 (7.6)
Mother's education				
Below junior college	4283 (21.8)	1548 (23.4)	1437 (22.1)	1298 (19.7)
Junior college/college	13254 (67.4)	4549 (68.9)	4411 (67.9)	4294 (65.3)
Above college	1407 (7.2)	452 (6.8)	463 (7.1)	492 (7.5)
Missing	729 (3.7)	53 (0.8)	183 (2.8)	493 (7.5)
Family income status				
Above average	5961 (30.3)	2083 (31.6)	1911 (29.4)	1967 (29.9)
Average	12680 (64.5)	4328 (65.6)	4262 (65.6)	4090 (62.2)
Below average	455 (2.3)	137 (2.1)	144 (2.2)	174 (2.6)
Missing	577 (2.9)	54 (0.8)	177 (2.7)	346 (5.3)
Rest 10 mins after 30-40 mins continuous eye use				
Always	8903 (45.2)	3407 (51.6)	2768 (42.6)	2728 (41.5)
Sometimes	9334 (47.5)	2811 (42.6)	3225 (49.7)	3298 (50.1)
Never	1245 (6.3)	331 (5.0)	377 (5.8)	537 (8.2)
Missing	191 (1.0)	53 (0.8)	124 (1.9)	14 (0.2)

Variables	Total, n (%)	2021	2022	2023
Over 40 cm distances from smartphone and tablet				
Always	12561 (63.9)	4646 (70.4)	3906 (60.1)	4009 (61.0)
Sometimes	5853 (29.8)	1573 (23.8)	2147 (33.1)	2133 (32.4)
Never	1078 (5.5)	330 (5.0)	328 (5.0)	420 (6.4)
Missing	181 (0.9)	53 (0.8)	113 (1.7)	15 (0.2)
One-punch, one-foot and one-inch				
Up to the standard	8080 (41.1)	2499 (37.9)	2742 (42.2)	2839 (43.2)
Not up to the standard	11413 (58.0)	4050 (61.4)	3639 (56.0)	3724 (56.6)
Missing	180 (0.9)	53 (0.8)	113 (1.7)	14 (0.2)
Piano player				
Yes	2518 (12.8)	917 (13.9)	815 (12.6)	786 (11.9)
No	16971 (86.3)	5632 (85.3)	5562 (85.7)	5777 (87.8)
Missing	184 (0.9)	53 (0.8)	117 (1.8)	14 (0.2)
Self-requirements on sitting and standing posture				
Constant keep	1720 (8.7)	648 (9.8)	533 (8.2)	539 (8.2)
Non-constant keep	17762 (90.3)	5898 (89.3)	5840 (89.9)	6024 (91.6)
Missing	191 (1.0)	56 (0.8)	121 (1.9)	14 (0.2)
Height adjustment on desk and seat at home				
No	5514 (28.0)	1851 (28.0)	1778 (27.4)	1885 (28.7)
Yes	13966 (71.0)	4695 (71.1)	4593 (70.7)	4678 (71.1)
Missing	193 (1.0)	56 (0.8)	123 (1.9)	14 (0.2)
Parents' view on the suitability of seat and desk for child				
Suitable	17473 (88.8)	5933 (89.9)	5725 (88.2)	5815 (88.4)
Not suitable	2006 (10.2)	613 (9.3)	645 (9.9)	748 (11.4)
Missing	194 (1.0)	56 (0.8)	124 (1.9)	14 (0.2)
Total PA (MET-min/w)	1053.00[372.00-2118.00]	1092.00[471.00-2070.00]	1053.00[360.00-2133.00]	1017.00[264.00-2133.00]
MVPA				
Sufficient	2135 (10.8)	678 (10.3)	729 (11.2)	728 (11.1)
Insufficient	17538 (89.2)	5924 (89.7)	5765 (88.8)	5849 (88.9)
Sedentary time (mins/d)	420.00[300.00-480.00]	360.00[240.00-480.00]	420.00[300.00-480.00]	450.00[320.00-540.00]
Sedentary time				
<4.5h/d	2177 (11.1)	919 (13.9)	680 (10.5)	578 (8.8)
≥4.5h/d	8549 (43.5)	2624 (39.8)	2838 (43.7)	3087 (46.9)
Missing	8947 (45.5)	3059 (46.3)	2976 (45.8)	2912 (44.3)

Table S3. Prevalence of SSP between boys and girls in total sample and stratified by education stage

Variables	Total	Boys	Girls
Total (n=19673)	223 (1.1)	85 (0.9)	138 (1.4)
Primary school			
Grade1	4 (0.1)	2 (0.1)	2 (0.1)
Grade2	4 (0.1)	3 (0.2)	1 (0.1)
Grade3	5 (0.1)	3 (0.2)	2 (0.1)
P-trend	0.73	0.661	0.998
Secondary school			
Grade6	76 (2.5)	37 (2.4)	39 (2.6)
Grade7	49 (1.6)	12 (0.8)	37 (2.5)
Grade8	85 (2.8)	28 (1.8)	57 (3.8)
P-trend	0.431	0.218	0.054
P-trend (Overall)	<0.001	<0.001	<0.001

Table S4 Total PA level and ST between groups over three years

Variables	Total	SSN	SSP	z	P
Overall					
Total PA (MET-min/w)	1053.00[372.00,2118.00]	1053.00[372.00,2118.00]	1122.00[418.88,2160.00]	0.392	0.695
Sedentary time (mins/d)	420.00[300.00,480.00]	420.00[300.00,480.00]	480.00[352.50,510.00]	2.223	0.026*
2021					
Total PA (MET-min/w)	1092.00[471.00,2070.00]	1090.00[466.50,2070.00]	1222.50[668.25,2112.75]	0.542	0.588
Sedentary time (mins/d)	360.00[240.00,480.00]	360.00[240.00,480.00]	407.50[360.00,480.00]	2.233	0.026*
2022					
Total PA (MET-min/w)	1053.00[360.00,2133.00]	1053.00[360.00,2133.00]	1380.00[346.50,2622.00]	0.697	0.486
Sedentary time (mins/d)	420.00[300.00,480.00]	420.00[300.00,480.00]	480.00[360.00,540.00]	2.282	0.023*
2023					
Total PA (MET-min/w)	1017.00[264.00,2133.00]	1017.00[264.00,2133.00]	975.00[315.50,1956.00]	0.340	0.734
Sedentary time (mins/d)	450.00[320.00,540.00]	450.00[322.50,540.00]	442.50[322.50,480.00]	0.372	0.710

Table S5. Estimated associations of Insufficient MVPA and excessive sedentary time with SSP in Secondary school (Model 1).

Variables	Model 1 (n=5,392)	
	OR (95%CI)	P
Secondary school		
MVPA		

Sufficient	ref.	
Insufficient	1.336 (0.801 to 2.227)	0.267
Sedentary time		
<4.5h/d	ref.	
≥4.5h/d	1.170 (0.609 to 2.249)	0.637

Note: Model 1 was adjusted by year.

Primary school was not included in the analysis due to the low prevalence of SSP.

Figure S1. Technical Guide for prevention and control of spinal curvature abnormality in children and adolescents.

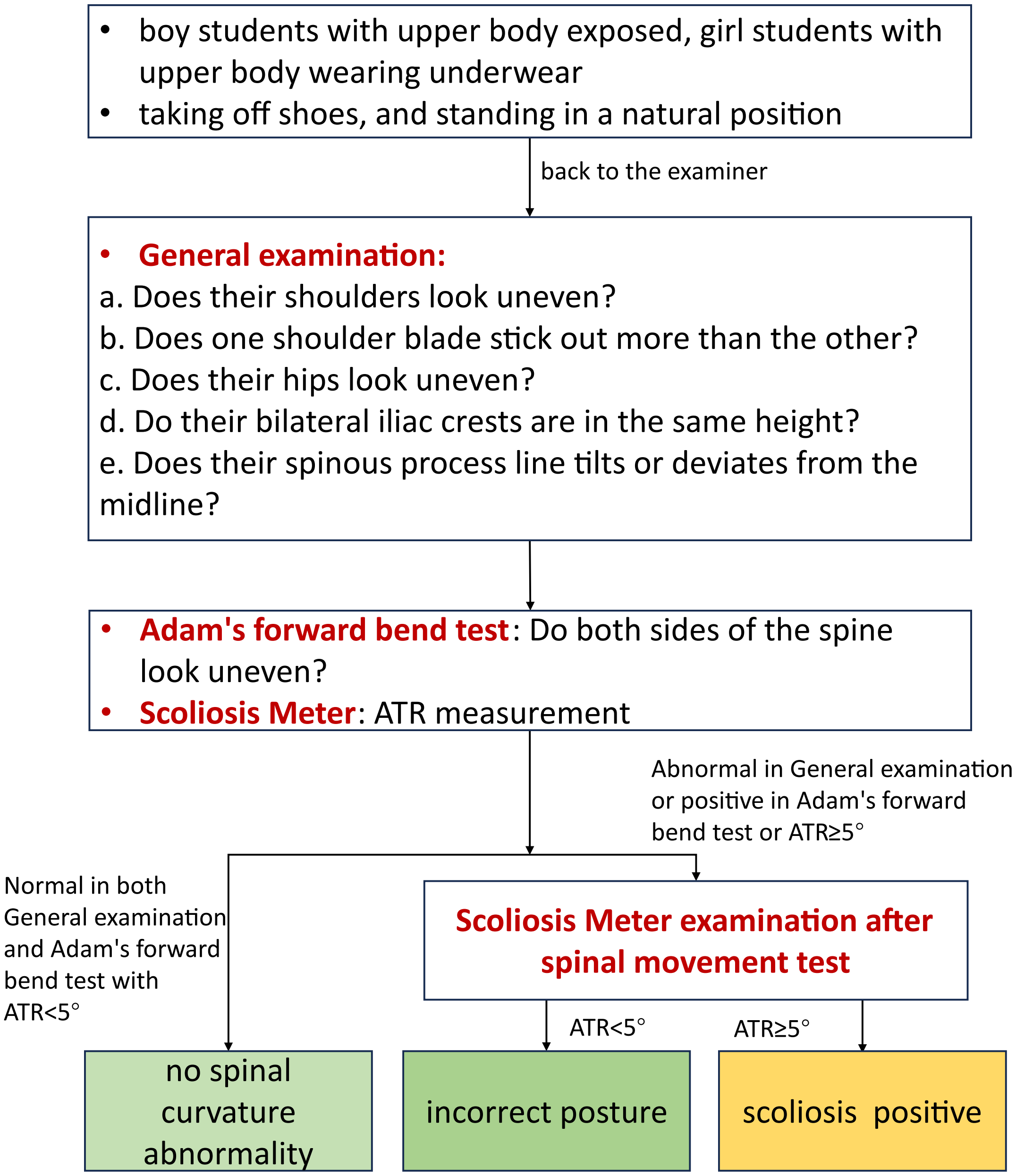

Figure S2. The correlation of age and sedentary time.

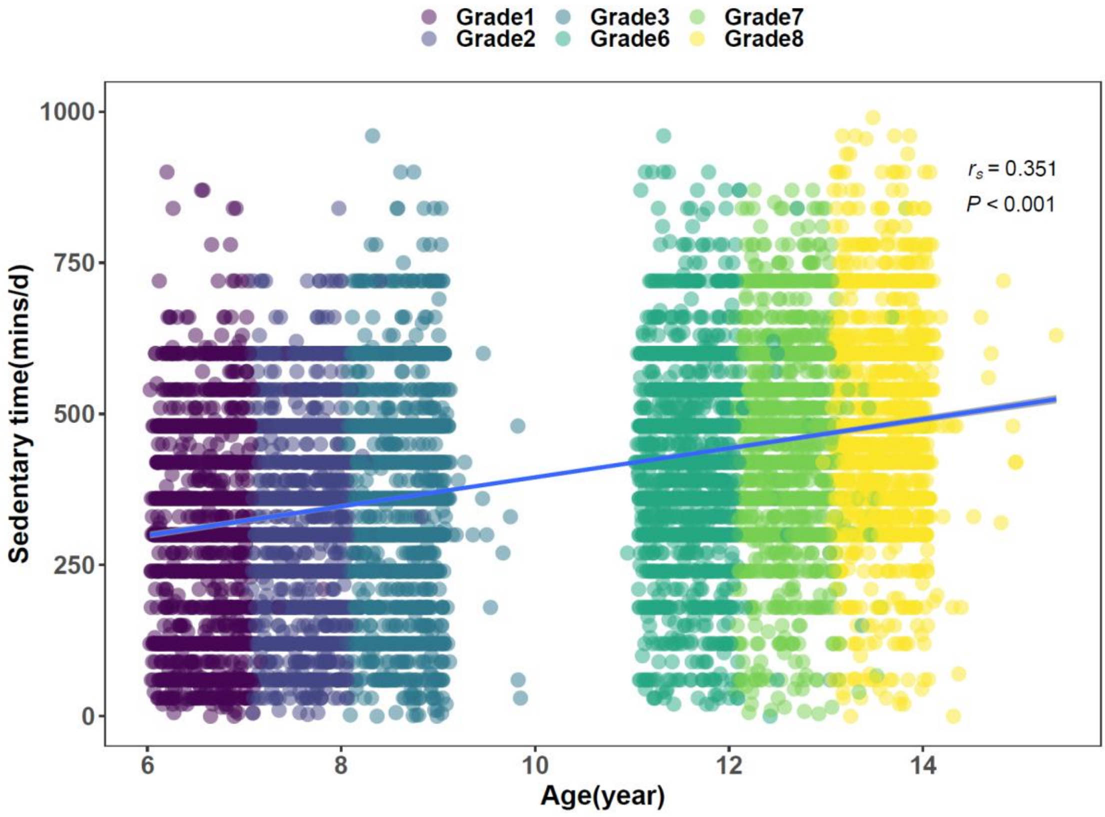

Figure S3. Distribution in levels of scoliosis among SSP students.

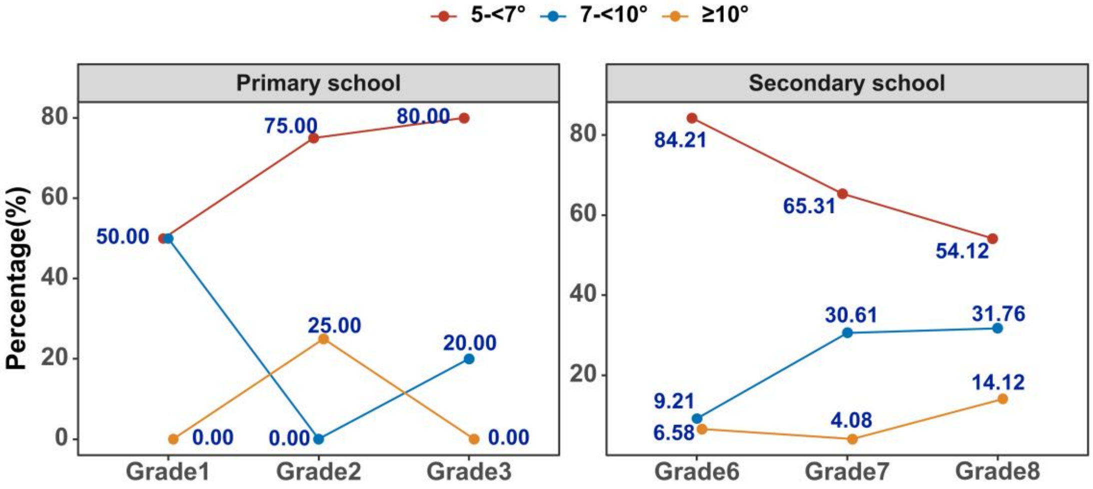

Supplement: Supplementary file 1 [file Data_Sheet_1.pdf]
